# Influence parental- and child-related factors on the acceptance of SARS-CoV-2 test methods in schools and daycare facilities

**DOI:** 10.3389/fpubh.2024.1264019

**Published:** 2024-07-18

**Authors:** Johanna K. Loy, Christian Kimmig, Simon Klos, Heidrun Lioba Wunram, Thorsten Langer, Eva Breitinger, Stephan Bender

**Affiliations:** ^1^University of Cologne, Faculty of Medicine and University Hospital Cologne, Department of Child and Adolescent Psychiatry, Psychosomatics and Psychotherapy, Cologne, Germany; ^2^Department of Pediatrics, University of Freiburg, Freiburg, Germany; ^3^Faculty of Medicine, School of Child and Adolescent Cognitive Behavior Therapy (AKiP), University Hospital Cologne, University of Cologne, Cologne, Germany; ^4^University of Cologne, Faculty of Medicine and University Hospital Cologne, Department of Pediatrics, Köln, Germany

**Keywords:** COVID-19, SARS-CoV-2, surveillance, test method, school, acceptance

## Abstract

**Introduction:**

Rapid testing for Severe Acute Respiratory Syndrome Coronavirus 2 (SARS-CoV-2) infections was an essential step in reducing the spread of the virus and monitoring pandemic development. Most mandatory standard pandemic testing in Germany has been performed in schools and daycare facilities. We investigated the influence of behavioral and attitudinal characteristics of children and caregivers on their acceptance of (i) antigen-based nasal swab rapid and (ii) oral saliva-based pooled Polymerase Chain Reaction (PCR) tests.

**Methods:**

Conducted through a cross-sectional survey between November and December 2021, with 1962 caregivers and 581 children/adolescents participating, the study evaluated the acceptability of each testing method on a six-point scale. Participants scored one test method conducted on their child at one of six levels with 1 and 6 denoting “excellent” (1) and “inadequate” (6), respectively. We considered demographic variables, vaccination status, child mental health (measured by the SDQ-questionnaire), and facility type (kindergarten, primary school, secondary school) as covariates.

**Results:**

Results reveal a preference for saliva-based PCR tests over nasal swabs by about one grade, particularly among parents of unvaccinated children, especially if their child expressed future vaccination reluctance. Testing acceptance was lower among children with mental health issues, primary school-aged, and those with less-educated parents. Perception of test accuracy and convenience influenced attitudes, favoring saliva-based PCR tests. Moreover, children with mental health issues felt less secure during testing.

**Discussion:**

To our knowledge, this is the first study to investigate the influence of different testing methods on testing acceptance for SARS-CoV-2 in children and caregivers. Our study identifies predictors of lower acceptance of public health surveillance measures and enables the development of educational programs on testing and vaccination tailored to the needs of specific target groups. Moreover, we demonstrate that test acceptance in vulnerable groups can be enhanced by careful choice of an appropriate testing method.

## 1 Introduction

The COVID-19 pandemic itself and its associated measures to protect the population have had far-reaching effects on the lives and well-being of children and adolescents worldwide (1, 2). Although children tend to have a milder clinical course of SARS-CoV-2 infections compared to adults, children can also become unwell either acutely or by developing Long Covid. Furthermore and in particular, the pandemic’s indirect effects on children’s socioeconomic, emotional, physical, and educational well-being and development have been immense (3). Children’s and young people’s daily lives have been affected by many pandemic-related public health measures – especially school and nursery closures.

### 1.1 COVID-19 transmission within educational institutions

The key argument for closing educational institutions was that transmission in such settings would play a significant role in driving up the incidence of SARS-CoV-2 infections (4). Closing these institutions was therefore a major public health strategy to reduce SARS-CoV-2 transmission and cut down its incidence. There were COVID-19-related school closures in 188 countries worldwide affecting over 1.5 billion students (5). For example, schools in Germany were closed for COVID-19-related reasons for a total of 38 weeks (as of June 2023) and approximately 55 million students in the USA could not physically attend classes for most of the 2020/2021 school year. However, according to current knowledge, the evidence supporting the effectiveness of school closures is mixed at best (6).

### 1.2 Consequences of closing educational institutions

Since educational institutions play a key role in ensuring children’s psychological and physical health as well as their socioeconomic prospects, their closure has exerted widespread and deep effects on the wellbeing of children and families.

First, given the paramount importance of schools for children’s educational development, a major potential long-term effect of the COVID-19 pandemic-caused school closures is the threat to their education (7). Compared to a typical school year, students have been returning to school with only 63–68% of the usual progress in reading and 37–50% in mathematics (8).

In addition, school closures have severe adverse effects on child health and well-being (9). These for example include malnutrition from having missed school meals, obesity due to lack of physical activity, and higher rates of mental health problems and intrafamilial abuse (10–14).

### 1.3 Targeted closures and avoiding school closures depend on regular testing

There is thus an inherent conflict of interest between limiting SARS-CoV-2 transmission on the one hand and the negative effects of school and daycare closures on the other. This is particularly true in light of the mixed evidence regarding the actual effects of school closures (6). One strategy to mitigate this dilemma is to implement targeted closures instead of comprehensive closures. This means that specific schools, daycare centers, classes, or groups would only close if SARS-CoV-2 cases are present or above a particular threshold.

The success of such targeted closures depends on rapid and reliable case detection so that closures can be implemented before widespread transmission occurs (15–18). In turn, detecting such cases relies on extensive, regular screening for SARS-CoV-2 infections. Consequently, governments implemented various testing regimes. In Germany, routine COVID-19 tests in schools and daycare centers became a key pillar of the strategy to keep as many educational institutions open as possible. The most common test options were nasal antigen tests (at home or on site; 20) and saliva-based pooled PCR tests (“lollipop-method”) on site (19). Overall sensitivity of antigen tests was reported at 63.2% in RT-PCR positive cases. In asymptomatic patients, sensitivity was 57.6% (20). All options entailed multiple tests per week.

Human behavior is a key component in “flattening the curve” and minimizing virus transmission (3). Regular testing of children in schools and daycare centers depends on high acceptance and compliance of children and parents (21). The uptake of voluntary testing in pilot projects has varied widely, from 1 to 68% (22). Understanding people’s attitudes toward testing may help to maximize the effectiveness of SARS-CoV-2 testing programs in educational settings and hence the success of the targeted-closures strategy. Moreover, understanding the emotional acceptance of routine screening in educational settings including that among different socioeconomic subgroups could yield insights into the acceptance of other public health interventions such as vaccine campaigns (23). Families with children in daycare and school settings represent an important group in the general population whose interests differ from those of adults without children or older adult people (24).

### 1.4 Factors influencing SARS-CoV-2 testing acceptance

Although weekly SARS-CoV-2 in-school testing was mandatory during the study period, questions remain regarding parents’ and children’s attitudes toward and their acceptance of such testing regimes. How well do people understand the importance of testing (25)? Do routine tests enable emotional acceptance by children and parents (26)? To what extent do socio-economic and demographic factors play a role in test acceptance (27–34)? Finally, is there a difference in the acceptance of different testing methods (antigen vs. PCR), maybe also due to their respective quality criteria (e.g., sensitivity)? COVID-19 incidence in the study period was high with 7-day incidences ranging between 91 per 100,000 in October to over 200 per 100,000 inhabitants in November and December 2021 (35). Regarding the perceived severity of the disease, it should be noted that until end of 2021 the Delta variant of COVID-19 was the most pervasive (36). Retrospective data indicate that the rate of hospitalizations with Delta was almost threefold higher compared to the Omicron variants. Therefore, the perceived severity of the disease might have been higher compared to subsequent periods of the pandemic.

Our sample was collected from November 11, 2021 to December 19, 2021. During the entire data collection, various vaccines were recommended and approved in Germany for children and adolescents aged 12 years and older. For children aged 5–12 years, the first vaccine was authorized during the period of data collection. According to data from the Robert-Koch Institute reported on December 20, 2021, 61.1% of children and adolescents aged 12 years and older had been vaccinated at least once; 50.6% were fully vaccinated. No specific data on the vaccination status of younger children were reported at this time nor were included in the above-mentioned percentages. First reported percentages from January 18, 2021 suggested a vaccination rate of 14.1% (vaccinated once) and 5.3% (fully vaccinated) for 5–11-year-old children (37).

Although there already is some evidence regarding the acceptability of SARS-CoV-2 testing in educational institutions, detailed studies including a broader set of further relevant aspects are scarce (38). To deepen this knowledge and to obtain a broader picture of associated factors, we examined the following:

1. What are different attitudes toward two SARS-CoV-2 testing methods [nasal antigen versus pooled PCR (19, 39)] in schools, and is there a relationship between different demographic factors (e.g., age, gender, parents’ educational status) and testing appraisal by parents and children. These factors are known to exert effects on testing hesitancy (29–34).
2. Is there a difference in testing appraisal between different types of childcare institutions (daycare, primary and secondary schools)?
3. Whether and how does the acceptance or rejection of a COVID-19 vaccine influence the appraisal of SARS-CoV-2 surveillance measures in schools? With this approach, we plan to expand upon the existing literature examining attitudes toward COVID-19 vaccinations (40–44). For example, Ali and colleagues (45) reviewed the global landscape of COVID-19 vaccine hesitancy, identifying governmental, healthcare system, population, and vaccine-related causes. They highlighted factors such as knowledge/awareness and social media influence, and proposed strategies to mitigate hesitancy at multiple levels, including structural, extrinsic, intrinsic, and other factors, aiming to facilitate vaccination efforts and combat hesitancy. To this end, we examined if doubting the vaccine’s benefit would be associated with a worse appraisal of testing as well.
4. How do mental health issues affect how surveillance is evaluated (46)? We examined this question due to the rise of mental health issues in children and adolescents during and after the pandemic (47, 48).

It should be noted that, as of December 2021, the German Standing Committee on Vaccination (STIKO) updated its COVID-19 vaccination recommendation, advising the vaccination of children aged 5–11 years with pre-existing conditions. In June 2021, the STIKO in Germany recommended COVID-19 vaccinations for adolescents aged 12–17, while in August 2021, they extended this recommendation to include COVID-19 vaccination for the general population within the same age group. As of now, there is no universal vaccination recommendation for individuals under 18 years of age in Germany.

## 2 Methods

### 2.1 Data

Data for this study refer to the COVID-19 pandemic situation in Germany and were collected between November 2021 and December 2021, a period where schools in Germany were operational, albeit with occasional adjustments such as the cancelation of mandatory attendance or the advancement of holiday breaks.

Study data were collected and managed using REDCap (electronic data capture tools), a web-based software platform (49, 50). Parents accordingly received links to online surveys for their participation. Links were distributed online and via schools, daycare facilities, clinics, and parent organizations. Children and adolescents aged 8 years and older were also provided with online links themselves.

Parents of children and adolescents aged 4–17 years in daycare facilities for children (pre−/playschools/kindergarten) as well as in primary and secondary schools in two German cities (Cologne and Freiburg) took part in this study. The sample is a convenience sample and therefore not representative of all of Germany. Recruitment was carried out by contacting school principals, parent organizations, and public city school councils who put up posters and involved their staff if they were willing to participate. Additionally, we put up study information in areas that are highly frequented by children and adolescents (e.g., pediatric emergency room). To minimize memory effects, the children’s last COVID-19 test had to have occurred within 7 days prior to the participation. There were no other inclusion or exclusion criteria. The subgroups answered similar questions as the participants from the parent sample. The main focus in this study is on the parent sample, examining factors influencing their evaluation and the impact of SARS-CoV-2 testing on families, additional analyses examining the adolescent sample are included as well. Consent of all participating parents and children/adolescents was obtained online via REDCap. Ethical approval was sought from the ethics committee of the university hospital in Cologne and in Freiburg (21–1,617).

### 2.2 Measures

#### 2.2.1 COVID-19 test methods

Subjects were asked to evaluate the last test for SARS-CoV-2 they had undergone within the previous 7 days, and whether they had experienced more than one test method (*n* = 256). As there were too few saliva antigen rapid tests, our analyses focused on comparing saliva-based PCR tests to nasal swab antigen rapid tests.

#### 2.2.2 Child mental health status

The Strength and Difficulties Questionnaire (SDQ) was used to assess child mental health status (51). The SDQ is a brief behavioral screening questionnaire adapted for 2–17-year-olds. It consists of emotional and behavioral screening that can, depending on the version employed, capture the perspective of children and young people, their parents, and teachers. There are five subscales in the long version of the SDQ (25 items), comprising subscales on emotional symptoms, conduct problems, hyperactivity/inattention, peer relationships problems, and prosocial behavior. We calculated the total difficulties score.

#### 2.2.3 Evaluation of COVID-19 tests

Subjects were asked to evaluate the COVID-19 tests according to the German school grading system ranging from 1 (best) to 6 (worst), with grades worse than 4 indicating failure in the class test in the school setting. This scaling was chosen because of its widespread use in various contexts in Germany and its suitability as a metric scale in statistical analyses. In emotional word lists according to the EWL-KJ (52), children reported their testing experience, and parents described how they thought their children experienced the test. Moreover, participants were asked about their attitudes toward SARS-CoV-2 testing via self-developed questionnaires on Likert scales ranging from 1 “false” and 2 “is probably not applicable” to 3 “is probably applicable” and 4 “true.”

We attached the respective questionnaires in the Supplementary material section.

### 2.3 Statistical analyses

First, we assessed potential differences in parent’s appraisal of SARS-CoV-2 testing in schools and daycare facilities depending on the test method and children’s age with a two-way ANOVA, with between-subjects factors being test method (swab antigen test/saliva-based PCR) and children’s age (kindergarten, 4–6 years/primary school, 6–10 years/secondary school, 10–17 years). The latter age categorization was used given the distinct educational environments in the corresponding groups. Notably, kindergarten settings have higher staff-to-child ratios and lack compulsory learning objectives, allowing more time for testing without impacting learning outcomes. However, younger children in kindergarten required more assistance during tests, resulting in fewer nasal swab antigen tests conducted in this setting within our sample. Thus, the initial analysis included only kindergarten data without further adjustments for confounding variables. We chose ANOVA since the dependent variable (school marks) could be treated linearly, despite a slightly skewed distribution toward better marks, which did not significantly affect the ANOVA’s robustness.

Second, we examined a general linear model to assess whether, how and which additional factors are associated with the appraisal of SARS-CoV-2 testing by parents. Due to the limited number of nasal swab tests in kindergartens, our analysis was confined to school-age children to ensure robust results. Linear predictors included age, gender, SDQ total score, attitude toward the COVID-19 vaccine and parents’ educational level, with educational level being dummy coded, SARS-CoV-2 testing method (saliva-based PCR test, nasal swab antigen test), and the vaccination status of the child (vaccinated or willing to be, no vaccination and unwilling, or unclear vaccination status).

Cities (Freiburg/Cologne) were also included into the model.

We hypothesized that parents with higher educational levels would rate COVID-19 surveillance more positively due to their emphasis on their children’s school education and better understanding of public health measures. Linear dummy coding was used to test this hypothesis, and additional regression models with different combinations of independent variables were calculated to examine the sensitivity of the model design.

Third, to examine how these factors are associated with the evaluation of SARS-CoV-2 testing, we applied an ordinal logit-model in a generalized linear model to establish how these factors covaried with specific aspects of SARS-CoV-2 testing as rated on the above-mentioned self-developed questionnaires (4 steps-Likert scale). Similarly, we calculated additional ordinal models with the same items of specific aspects of SARS-CoV-2 testing using children’s evaluation of SARS-CoV-2 testing, parents’ SDQ total score, and children’s vaccination status as dependent variables.

Despite the right-skewed distribution of the dependent variables (e.g., grade rated by parents: skewness = 1.43; kurtosis = 4.31), we opted to retain the original numerical values to preserve comprehensive data representation. Simplifying these ordinal variables might lead to significant information loss and reduced granularity, impacting the interpretation of our results and implications for policy decisions.

All analyses were performed with Statistica 13, TIBCO Software Inc.

## 3 Results

### 3.1 Participants

A sample of 1962 parents (371 male, 1,589 female, 2 diverse/non-binary; mean age 43.0; of 961 boys, 997 girls, and 4 diverse/non-binary children; mean age 8.6 years) participated in the study enabling complete data sets.

Additionally, we collected a sample of 581 children and adolescents (205 male, 372 female, 4 diverse/non-binary, mean age 13.1 years) which is part of further exploratory analysis.

Details on our sample’s age distribution can be found in Table 1. This includes a summary of the proportion of parents who (strongly) agreed with the corresponding statements to facilitate the assessment of the overall acceptance and the interpretation of the results.

**Table 1:** Descriptive statistics on the age distribution of children of our parents and children/adolescents samples.

Parents sample			Children Sample	
Age of their children (years)	*n*	%	Age (years)	*n*
4–5	520	26.50	8–9	66
6–7	349	17.79	10–11	105
8–9	310	15.80	12–13	122
10–11	330	16.82	14–15	161
12–13	230	11.72	16–17	127
14–15	149	7.59		
16–17	74	3.77		

Sex of their children	*n*	%	Sex	*n*	%
Male	961	48.98	Male	205	35.28
Female	997	50.82	Female	372	64.03
Diverse	4	0.2	Diverse	4	0.69

Vaccination status	*n*	%	Vaccination status	*n*	%
Yes	350	17.84	Yes	205	35.28
No	1,590	81.04	No	372	64.03
No answer	22	1.12	No answer	4	0.69

Test method	*n*	%	Vaccination status	*n*	%
Lolli PCR	1,416	72.17	Lolli PCR	396	68.16
Nasal swab antigen	503	25.64	Nasal swab antigen	177	30.46
Saliva-antigen	43	2.19	Saliva-antigen	8	1.38

Location	*n*	*%*	Location	*n*	%
Cologne	904	46.08	Cologne	202	34.77
Freiburg	1,058	53.92	Freiburg	379	65.23
	Total: 1962			Total: 581	

Non-binary subjects were excluded from further statistical analyses because of their low number. Participating parents on average had a relatively high educational level (primary/middle school *n* = 274; “Fach−/Abitur”/high school *n* = 533; university *n* = 1,149). Two parents with no school qualification were excluded due to their low number (Table 1).

A total of *n* = 440 children of the participating parents were unvaccinated and did not want to be vaccinated in the future according to their parents. In sum, *n* = 994 children were either vaccinated, or their parents reported their intention to have their child vaccinated as soon as a recommended vaccination became available. *N* = 524 parents provided no information about their child’s current vaccination status or their vaccination intention in the future. The children of *n* = 1,863 parents had never had a COVID-19 infection, *n* = 81 had, *n* = 14 did not give information about whether their child had had a COVID-19 infection. Children of 1,414 parents underwent saliva-based PCR tests, 503 antigen rapid tests via nasal swab (thereof 501 with ratings for the test method), and 43 antigen rapid tests based on saliva.

The proportion of (strong) agreement with the statements on average was 40%, it varied between 7% for “My child feels insecure when performing COVID-19 tests.” and 70% for “The COVID-19 test is usually over quickly for my child.”

### 3.2 COVID-19 test-method appraisal and influencing factors

**Figure 1:**
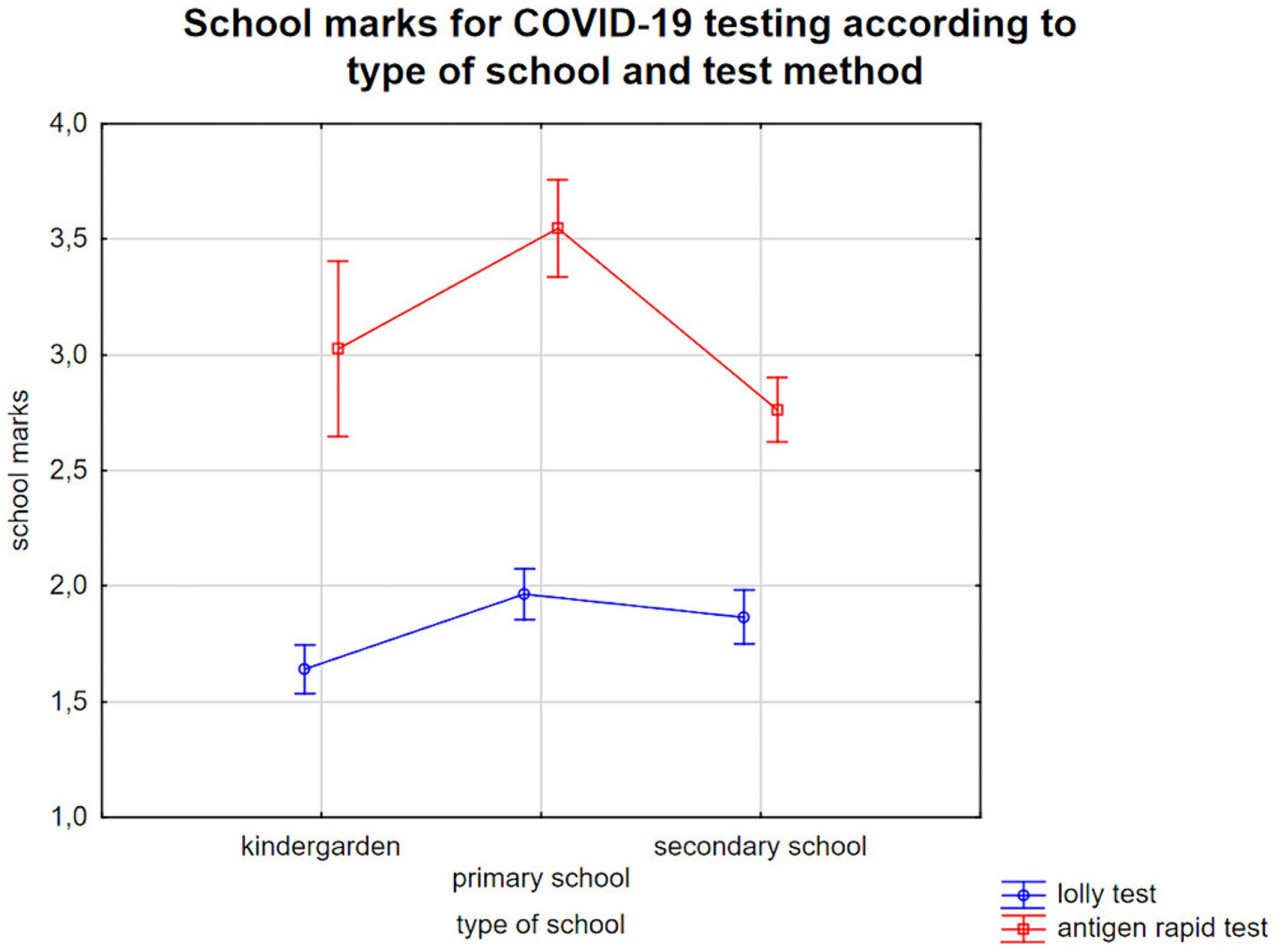
School grades (1—very good, 2—good, 3—satisfactory, 4—sufficient, 5—insufficient, 6—poor) for COVID-19 testing according to the child care setting and the children/adolescents’ age (kindergarten: 4–6 years, primary school: 6–10 years, secondary school: 10–17 years).

First, the ANOVA revealed that saliva-based PCR testing was consistently and significantly rated better across all age groups [main effect test method *F*(1, 1,862) = 233.7; *p* < 0.0001; η*_p_*^2^ = 0.11]. We also noted a statistically significant interaction between age and test-method [*F*(2, 1,862) = 10.6; *p* < 0.0001, η*_p_*^2^ = 0.01], indicating that only parents of primary school children rated antigen tests worse than did parents of adolescents in secondary school (Scheffe *post-hoc* test *p* < 0.0001). The parents of kindergarten children rated the tests better than those of children in primary school. That finding was significant for saliva-based PCR tests (*p* = 0.004), but not for the few antigen tests performed in kindergarten (*p* = 0.35) (Figure 1).

Due to the small number saliva antigen tests were omitted from these analyses as the added value of the additional information would be low. Allocation to one of the other two groups, i.e., testing salivary sampling versus swab sampling, could lead to a distortion of the results. Instead, Supplementary Table S1 provides a descriptive comparison of the parent ratings for the different test methods.

**Figure 2:**
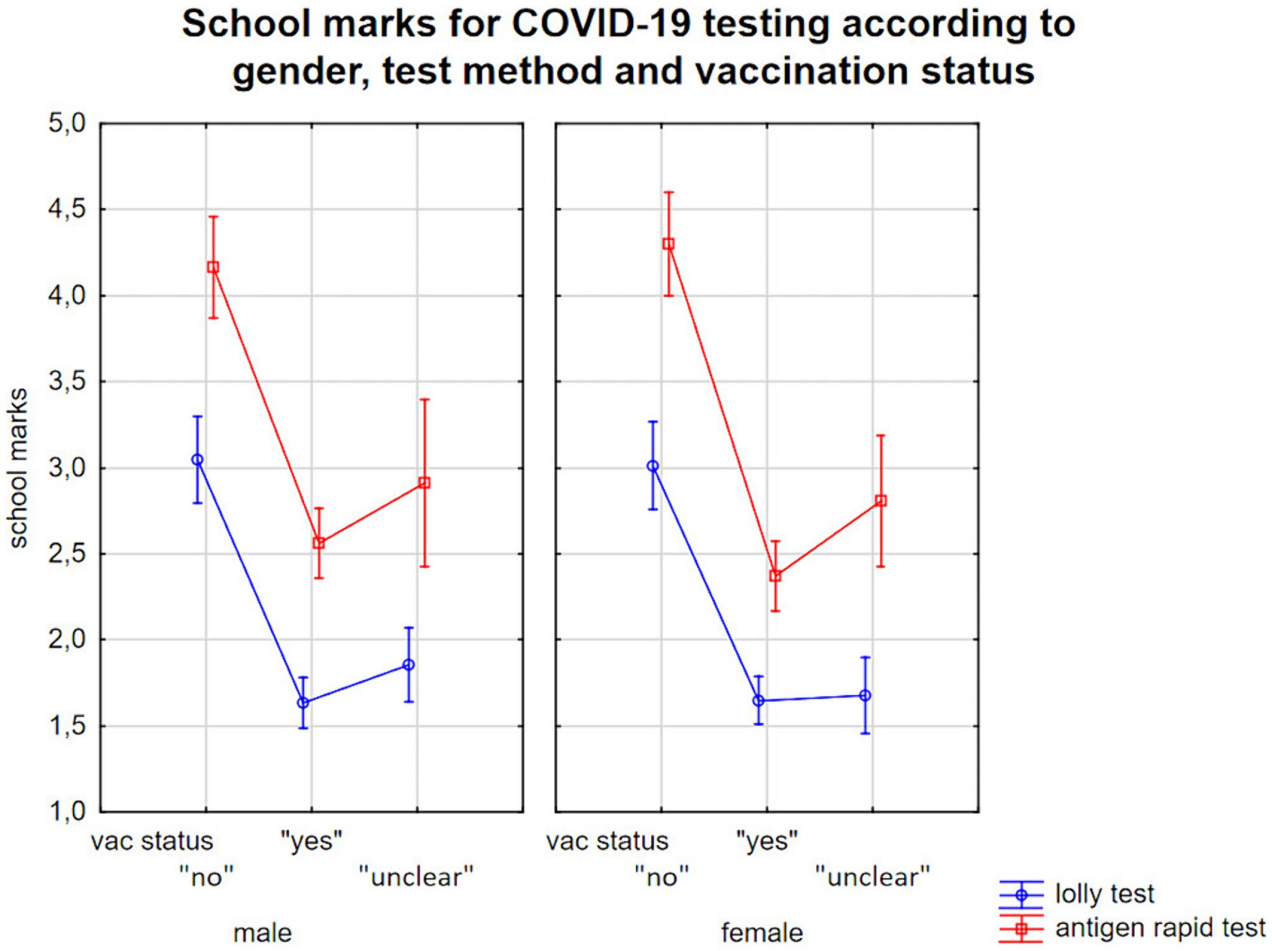
School grades for COVID-19 tests according to test method, children’s gender and vaccination status/parents report on their children’s’ willingness to be vaccinated.

Second, our general linear model analyses showed that the categorical factors gender, SARS-CoV-2 testing method, vaccination status of the children and linear predictors age, mental health status and the parents’ pseudo-coded educational level explained 34% of the variance (corrected R^2^) in a highly significant model [*F*(7, 1,307) = 97.1; *p* < 0.0001]. All factors except for age and gender showed highly significant influences on the parents’ evaluation of SARS-CoV-2 testing by school grades (Figure 2). In contrast, the additional factor “region” (Cologne or Freiburg) did not enter the final model as it had no significant effect [*F*(1, 1,306) = 0.55; *p* = 0.46] and did not increase the explained variance. Mean values and standard errors are presented in Table 2, further details are provided in Table 3.

**Table 2:** Mean values and standard errors (SE) assessing COVID-19 tests by parents (school grades ranging from 1 to 6).

	Vac status	Saliva-based PCR test			Nasal swab antigen test		
		*N*	Mean	*SE*	*N*	Mean	*SE*
Male	“No”	82	3.05	0.13	60	4.17	0.15
	“Yes”	242	1.63	0.07	123	2.56	0.10
	“Unclear”	110	1.85	0.11	22	2.91	0.25
Female	“No”	78	3.01	0.13	57	4.30	0.15
	“Yes”	275	1.65	0.07	127	2.38	0.10
	“Unclear”	105	1.68	0.11	36	2.81	0.20

Vac Status = vaccination status; no = unvaccinated/no vaccination information and unwilling to be vaccinated, yes = vaccinated or willing to be, unclear = no information provided about vaccination or vaccination intention.

**Table 3:** Effects of age, gender, SDQ total score, parents’ educational level, vaccination status/hesitancy and test method on COVID-19 test ratings—regression coefficients (*n* = 1,315).

Effect	Parameter	*SE*	*p*	95% CI	
				Lower bound	Higher bound
Age	−0.01	0.01	0.91	−0.02	0.02
Gender	0.01	0.03	0.85	−0.06	0.07
SDQ total	0.04	0.01	0.001*	0.05	0.05
Educational level	−0.11	0.04	0.001*	−0.20	−0.02
Vaccination status (unvaccinated vs. vaccinated)	0.87	0.05	0.001*	0.76	0.98
vaccination status unvaccinated vs. unclear status)	−0.52	0.05	0.001*	−0.61	−0.43
COVID-19 test method	−0.47	0.03	0.001*	−0.53	−0.40

* statistically significant, *p* < 0.05.

For the full model, R^2^ was 0,342 and adjusted R^2^ was 0,339. Results of the additional models with different combinations of independent variables confirmed the results. Corresponding results including R^2^ and adjusted R^2^ can be found in Supplementary Tables S2–S7.

We found a strong association between vaccination status and appraisal of SARS-CoV-2 testing [*F*(2, 1,306) = 135.6; *p* < 0.001], with parents with unvaccinated children and unwilling to be vaccinated rating COVID-19 tests about one and a half school grades lower than parents whose children were vaccinated or who reported that their child wanted to be vaccinated. Higher SDQ scores, i.e., more mental health issues, also predicted a worse COVID-19 test evaluation [*F*(1, 1,306) = 38.8; *p* < 0.001], with about 10 SDQ-total score points triggering an about half-grade worse evaluation (*cf.* Table 3). Finally, we observed a small effect of parent’s educational level [*F*(1, 1,306) = 6.0; *p* = 0.01], with parents with no high-school degree evaluating SARS-CoV-2 testing about 0.2 evaluation scores lower than parents with a university degree (*cf.* Table 3).

Third, in an ordinal logit-model we showed that parents based their rating mainly on a correct test result (Wald statistic = 142.5; *p* < 0.001) and on whether the tests helped that their child could attend school safely (Wald statistic = 40.4; *p* < 0.001). They also worried about whether their child felt comfortable with the test but to a lesser extent.

We tested additional ordinal logit-models exploratorily. Contrary to their parents, children and adolescents placed stronger emphasis on the test’s convenience and painlessness (Wald statistic = 29.4; *p* < 0.001). Among them, this was the most important factor. Details are found in Table 4.

**Table 4:** Ordinal logit model—prediction of COVID-19 test evaluations (school grades) by parents/children’s attitudes toward SARS-CoV-2 testing (*n* = 1962/*n* = 581).

		Coefficient	*P > |z|*	[95% CI]
Parents				
1	“The COVID-19 test is easy for my child.”	0.159	0.123	−0.043; 0.361
2	“My child feels insecure when performing COVID-19 tests.”	0.044	0.621	−0.130; 0.217
3	“The COVID-19 test is usually over quickly for my child.”	0.091	0.288	−0.076; 0.258
4	**“I think my child loses a lot of time performing COVID-19 tests”**	**0.164**	**0.006**	**0.048; 0.281**
5	**“I think it’s a good idea, and consent to my child’s being tested for COVID-19 at school/in kindergarten.”**	**−0.427**	**<0.001**	**−0.589; −0.265**
6	**“I think my child likes the COVID-19 test.”**	**0.314**	**<0.001**	**0.168; 0.459**
7	“I have to wait too long for the test result.”	0.105	0.048	0.001; 0.209
8	**“I believe the COVID-19 test result is accurate.”**	**−0.715**	**<0.001**	**−0.851; −0.580**
9	“My child finds the COVID-19 test disgusting.”	−0.159	0.051	−0.320; 0.001
10	“My child does not find the COVID-19 test unpleasant.”	0.052	0.373	−0.063; 0.168
11	“My child dislikes the COVID-19 test (e.g., it hurts).”	0.090	0.226	−0.056; 0.235
12	“My child is embarrassed to do the COVID-19 test together with its class/group.”	−0.066	0.388	−0.217; 0.084
13	**“COVID-19 testing helps my child attend school safely.”**	**−0.366**	**<0.001**	**−0.520; −0.211**
14	“It helps my child that COVID-19 test result from school makes it easier to engage in leisure activities.”	−0.017	0.727	−0.113; 0.079
15	“My child would feel embarrassed to get a positive test result in the classroom and have to be taken home by their parents.”	0.040	0.409	−0.055; 0.134
16	**“My child prefers/would like to get the result on the next day (and not in the classroom).”**	−**0.117**	**0.010**	**−0.206; −0.028**
17	**“Which grade would your child give the current test method in school or daycare?”**	**1.882**	**<0.001**	**1.725; 2.039**
Children				
1	“The COVID-19 test is easy for me.”	0.003	0.986	−0.323; 0.329
2	“I feel insecure when performing COVID-19 tests.”	0.105	0.446	−0.165; 0.375
3	“The COVID-19 test is usually over quickly.”	−0.122	0.372	−0.389; 0.145
**4**	**“I think we lose a lot of time performing COVID-19 tests.”**	**0.395**	**<0.001**	**0.203; 0.587**
**5**	**“I think it’s a good idea and agree to perform this COVID-19 test at school.”**	**−0.651**	**<0.001**	−**0.917; −0.385**
6	“I have to wait too long for the test result.”	0.069	0.437	−0.106; 0.244
**7**	**“I believe the COVID-19 test result is accurate.”**	**−0.568**	**<0.001**	−**0.815; −0.322**
8	“I find the COVID-19 test disgusting.”	0.146	0.239	−0.097; 0.390
9	“I do not find the COVID-19 test stressful.”	0.052	0.539	−0.114; 0.218
**10**	**“I find/found the COVID-19 test unpleasant.”**	**0.630**	**<0.001**	**0.411; 0.850**
11	“I’m embarrassed to do the COVID-19 test together with my class/group.”	0.178	0,0.186	−0.086; 0.441
**12**	**“I think the COVID-19 tests help me attend school safely so that I do not have to do home-schooling.”**	**−0.662**	**<0.001**	**−0.935; −0.388**
13	“It helps that COVID-19 test result from school makes it easier for me to engage in leisure activities.”	−0.136	0.083	−0.291; 0.018
14	“I would feel embarrassed to get a positive test result in the classroom and have to be taken home by my parents.”	0.045	0.573	−0.111; 0.200
15	“I prefer/would like to get the result on the next day (and not in the classroom).”	−0.076	0.296	−0.219; 0.067

Bold text indicates statistically significant predictors, *p* < 0.05.

The SDQ total score was predicted by items referring to the children’s insecurity or irritability, how easy or difficult the test itself would be, as well as whether it was perceived as a burden by the child (Table 5). This score reflects the relationship between children’s testing experience, irritability, and an increased burden of mental health problems.

**Table 5:** Prediction of parents-rated SDQ total score by parental attitudes toward SARS-CoV-2 testing.

		Coefficient	*P > |z|*	[95% CI]
1	“The COVID-19 test is easy for my child.”	−0.235	0.006	−0.402; −0.068
2	“My child feels insecure when performing COVID-19 tests.”	0.25	<0.001	0.109; 0.390
3	“The COVID-19 test is usually over quickly for my child.”	−0.101	0.153	−0.240; 0.038
4	“I think my child loses a lot of time performing COVID-19 tests”	−0.01	0.841	−0.109; 0.089
5	“I think it’s a good idea, and consent to my child’s being tested for COVID-19 at school/in kindergarten.”	−0.032	0.644	−0.166; 0.103
6	“I think my child likes the COVID-19 test.”	−0.029	0.631	−0.147; 0.089
7	“I have to wait too long for the test result.”	0.058	0.185	−0.028; 0.144
8	“I believe the COVID-19 test result is accurate.”	−0.07	0.213	−0.179; 0.040
9	“My child finds the COVID-19 test disgusting.”	−0.064	0.363	−0.203; 0.074
10	“My child does not find the COVID-19 test unpleasant.”	−0.138	0.005	−0.235; −0.041
11	“My child dislikes the COVID-19 test (e.g., it hurts).”	0.053	0.396	−0.070; 0.176
12	“My child is embarrassed to do the COVID-19 test together with its class/group.”	0.17	0.010	0.041; 0.299
13	“COVID-19 testing helps my child attend school safely.”	0.105	0.120	−0.027; 0.237
14	“It helps my child that COVID-19 test result from school makes it easier to engage in leisure activities.”	−0.114	0.004	−0.193; −0.036
15	“My child would feel embarrassed to get a positive test result in the classroom and have to be taken home by their parents.”	0.108	0.007	0.030; 0.187
16	“My child prefers/would like to get the result on the next day (and not in the classroom).”	0.03	0.408	−0.042; 0.103
17	“Which grade would your child give the current test method in school or daycare?”	0.021	0.700	−0.087; 0.129

Finally, we found that the vaccination status was associated with the parent’s willingness to have their child undergo COVID-19 tests at school, and whether they believed such tests would help their child attend school safely (Table 6).

**Table 6:** Prediction of vaccination status by parents’ attitudes toward SARS-CoV-2 testing.

		Coefficient	*P > |z|*	[95% CI]
1	“The COVID-19 test is easy for my child.”	−0.327	0.038	−0.636; −0.018
2	“My child feels insecure when performing COVID-19 tests.”	0.222	0.083	−0.029; 0.472
3	“The COVID-19 test is usually over quickly for my child.”	−0.032	0.807	−0.286; 0.222
4	“I think my child loses a lot of time performing COVID-19 tests”	−0.076	0.428	−0.262; 0.111
5	“I think it’s a good idea, and consent to my child’s being tested for COVID-19 at school/in kindergarten.”	−0.745	<0.001	−0.970; −0.520
6	“I think my child likes the COVID-19 test.”	−0.345	0.002	−0.560; −0.129
7	“I have to wait too long for the test result.”	−0.126	0.154	−0.300; 0.047
8	“I believe the COVID-19 test result is accurate.”	−0.163	0.111	−0.364; 0.038
9	“My child finds the COVID-19 test disgusting.”	0.296	0.018	0.051; 0.541
10	“My child does not find the COVID-19 test unpleasant.”	−0.081	0.397	−0.268; 0.106
11	“My child dislikes the COVID-19 test (e.g., it hurts).”	−0.153	0.217	−0.396; 0.090
12	“My child is embarrassed to do the COVID-19 test together with its class/group.”	0.007	0.955	−0.234; 0.248
13	“COVID-19 testing helps my child attend school safely.”	−0.417	<0.001	−0.642; −0.191
14	“It helps my child that COVID-19 test result from school makes it easier to engage in leisure activities.”	0.308	<0.001	0.138; 0.479
15	“My child would feel embarrassed to get a positive test result in the classroom and have to be taken home by their parents.”	0.008	0.915	−0.140; 0.156
16	“My child prefers/would like to get the result on the next day (and not in the classroom).”	0.12	0.096	−0.021; 0.262
17	“Which grade would your child give the current test method in school or daycare?”	−0.034	0.74	−0.233; 0.165

### 3.3 Child mental health status

The parent-rated SDQ revealed a mean total score of 8.9 ± 5.9 in this non-clinical sample. *N* = 1,692 parents rated their children in the 90% “normal” range according to German norms (53), while *n* = 264 parents (13.5%) rated their children as having more mental health problems (compared to 10% in the normative sample). The 8.9 mean value was significantly higher (*p* < 0.001) than the mean value in normative samples in Germany or in the USA before the pandemic (53, 54), indicating a slight increase in overall mental health issues associated with the pandemic in the examined sample.

## 4 Discussion

Several studies have already investigated the acceptance of COVID-19 tests by using different samples (e.g., parents, students, school staff) and methodological approaches (online surveys, qualitative interviews, focus groups, experimental designs) (38, 55–60). Our study is primarily related to those studies that either included the perspective of parents or of parents/school staff and children (38, 57–60). In terms of the evaluation of saliva-based COVID-19 tests, other studies indicated a high level of acceptance and feasibility among parents and their children (59). Only one study specifically aimed to provide a comparison of different COVID-19 test methods (nasal swab testing vs. salvia-based testing) and included the perspective of children and adolescents (38). Within the group of children and adolescents, this study showed balanced evaluations between the two different test methods in terms of preference. Reasons for favoring the nasal swab included that it is quicker and easier. Reasons for favoring saliva-based tests included that it was more fun and easier. However, the study does have limitations in terms of generalizability due to its small sample size (*N* = 135 with *n* = 67 students) and the fact that no parent ratings were collected. In the adult population, there are already larger studies aimed at comparing acceptance ratings of different COVID-19 test methods (56). Our study provided substantive additional knowledge by analyzing the acceptance of two different testing methods and potential risk factors in large samples, including the experiences of parents and their children.

Summarizing our main findings, we discovered:

1. overall better acceptance of saliva-based PCR tests rather than rapid nasal swab antigen tests
2. less acceptance of SARS-CoV-2 testing by parents of children in primary school compared to those with children in kindergarten and secondary school
3. an association between SARS-CoV-2 testing acceptance, test methods, and vaccination status: results reveal a preference for saliva-based PCR tests over nasal swabs by about one grade, particularly among parents of unvaccinated children and if their child expressed future vaccination reluctance
4. a negative association between mental health problems and SARS-CoV-2 testing acceptance.

### 4.1 Saliva-based PCR testing versus nasal swab antigen testing

Overall, our research suggests better acceptance of saliva-based PCR testing compared to nasal swab antigen testing among parents of children across all three age groups. This is in line with prior studies that also reported a high acceptance of PCR saliva-based testing in children (19, 39, 61, 62). Similarly, combined throat and nasal swabs have also been described as a feasible alternative down to 4 years of age in Western Australia (63).

The saliva-based method’s test ratings were on average 1.5 evaluation scores higher than those of nasal swab antigen tests. Compared to antigen tests, parents valued the PCR tests’ greater accuracy, while children found the test to be less uncomfortable. It is evident from our results that the comfort level of the test is highly relevant for children and adolescents, underlining the importance of individual testing experience in this population. Therefore, gaining insight into the children’s experiences with the tests is vital and needs to be considered to develop a child-centered approach.

The delay in receiving the results on the next morning was rated less important. This finding is in line with studies reporting perceived test-correctness as an important factor (26, 64, 65).

Importantly, our results regarding the mandatory serial testing of asymptomatic children at school differ from how self-collected nasal swab antigen tests collected by symptomatic children and adolescents actively cared for by the healthcare system were rated in France (66), with the latter rating nasal swabs more positively. The same seems to apply to adult subjects in Germany (67). Together, these findings suggest benefits of voluntary surveillance measures and imply that in case of mandatory testing the most reliable and convenient test method should be employed (saliva-based PCR tests). Other studies also showed that the highest long-term participation rates in school surveillance settings were obtained using saliva-based testing (68) and biweekly saliva testing of at least 50% of children and staff has been recommended to limit secondary infections (69). Such rates seem attainable through voluntary saliva-based PCR tests, also due to perceived high reliability of the results. Alternatives to saliva-based PCR tests have been proposed, such as gargling at home and pooling probes at school (70, 71).

### 4.2 Understanding the test-taking population and their motivation

#### 4.2.1 The influence of age on test ratings

Age has been shown to be an important predictor for the attitude and acceptance of both COVID-19 vaccination and testing (30–32, 72). While this might reflect age-associated differences in attitude toward the pandemic situation in adults, it could indicate a shortage of resources to deal with test requirements relative to the available support by adult caregivers (teachers, educational staff) in children and adolescents. In contrast to schools and kindergartens, the acceptability of all test methods has been suggested to be high in university settings (55).

Consequently, primary school children might need more support while testing compared to older individuals, as they might be already burdened by the requirements of learning at school (higher demands but fewer adult caregivers available than in pre-school children’s daycare facilities).

#### 4.2.2 Vaccination willingness and association with test ratings (school grade)

Vaccination status and children’s willingness to be vaccinated were important predictors of how surveillance was rated by the parents, with rejecting vaccination being associated with lower acceptance of COVID-19 surveillance. In general, we observed many more families who categorically either favored or rejected COVID-19 measures than families who harbored specific concerns about the vaccination and would readily agree to serial testing to protect their child. For future vaccination and surveillance strategies, information campaigns should consider specific concerns as well as a general mistrust toward public health measures. In the group of parents reporting that their children were unwilling to be vaccinated, the antigen test ratings dropped to a mean level of a grade 4 (on the scale from 1 to 6). Considering the variance in the other group’s ratings, this can be deemed a considerable difference in test perception and suggests that antigen testing could be an insufficient surveillance strategy in this group. In comparison, screening via saliva-based PCR tests did not dissolve the effect of general attitudes toward COVID-19 public health measures, but appeared to mitigate this effect and elevate ratings to the “satisfactory” level (grade 3). Therefore, this test method might be an especially important factor in raising the acceptance of test strategies in vulnerable groups and also emphasizes a potential benefit of adapting public health measures to specific target groups.

#### 4.2.3 Testing acceptance and mental health problems

Our study found that children and adolescents with mental health issues were more likely to reject COVID-19 surveillance measures in public schools and daycare facilities compared to those without such issues. Their reluctance toward testing methods correlated with higher levels of anxiety or insecurity, underscoring the need for tailored support and reassurance during virologic tests. Screening these vulnerable groups for concerns about testing could prove beneficial in alleviating apprehensions.

The pandemic itself has exacerbated anxiety and mental health symptoms in children and adolescents (47, 48, 73), possibly due to reduced social contact and physical activity (74). Thus, specific assistance for vulnerable groups is crucial in shaping effective public health measures (47, 48, 73). While more anxious adults are generally more accepting of testing (75), parents of children with behavioral or mental health issues in our study reported more negative perceptions and greater difficulties with testing. Notably, we did not find an association between mental health issues and a negative attitude toward pandemic public health measures overall, unlike findings in samples of depressed individuals (76).

#### 4.2.4 The influence of parental educational background

Consistent with previous research (26, 32), our study revealed a modest impact of parental educational background on perceptions of COVID-19 tests. Parents with higher educational attainment may have better access to information and tend to weigh the benefits and costs of SARS-CoV-2 testing differently. Conversely, individuals with lower educational backgrounds may rely more on information from family and friends rather than scientific sources (77). However, corresponding findings have been inconsistent (78–80). Thus, each country’s specific conditions have to be considered and examined separately.

### 4.3 Limitations

When interpreting the results, some limitations need to be considered. Convenience sampling was used, which is known to be limited by potential selection bias and external validity of findings. This may hinder the ability to make causal inferences. Although we were unable to collect data from a representative sample for German society, our large cohorts and the absence of effects of the place of residence suggest that valid conclusions can be drawn and somewhat generalized. We still need to confirm our results with independent samples, as we cannot exclude the possibility that certain parent groups were more likely to respond to our survey than others (81). There is evidence of local differences among adult subjects in different regions (UK, China) (82). Though public health measures differ somewhat among the German states of North Rhine-Westphalia (Cologne) and Baden-Württemberg (Freiburg), the environmental variables between Cologne and Freiburg seem to have been quite homogeneous.

As only a tiny subgroup of children had experienced both sampling methods and were asked to evaluate the last sampling method employed, we were unable to conduct within-subject comparisons of the two methods. However, our results converge with findings of within-subject comparisons in smaller samples, showing a preference for saliva sampling over nasopharyngeal swabs (83).

In addition to distributing the online survey to parents and children via teachers and educators, we advertised our study via notice boards in schools and daycare centers. It is therefore possible that people may have participated who do not belong to the intended sample group, which could have compromised the validity of our results. However, any biases, if evident, should not be significant thanks to our large sample.

An additional limitation is that we did not incorporated the latest epidemiologic data in our analyses. The number of infection rates and the current pandemic situation might have a biasing effect on how people rate COVID-19 tests. Any proposals we have for future testing should be derived after considering the most recent epidemiologic data.

## 5 Conclusion

In this study, we observed wide variability in testing acceptance across different demographic groups and factors. We identified parental educational level, children’s age, mental health status, and vaccination willingness as significant factors influencing acceptance of COVID-19 surveillance measures. Saliva-based PCR testing emerged as a preferred method, particularly for serial testing in schools and daycare facilities, potentially enhancing acceptance among vulnerable groups. These findings provide valuable insights for policymakers when formulating future testing strategies. While our results need replication due to our non-representative sample, the implications from this large cohort study should be carefully considered in shaping future public health policies.

## 6 Propositions for public health testing strategies

Our study aims to provide precise and actionable recommendations for future pandemic testing strategies, applicable to COVID-19 or other infectious diseases irrespective of specific virus variants. Building on the WHO framework for vaccination behavioral and social drivers (69), alongside our comprehensive analysis and existing literature, we propose the following recommendations:

1. Choosing the right test method

*Voluntary testing:* Whenever feasible, offering voluntary testing options enhances individual autonomy and ownership, potentially improving test acceptance rates.

*Saliva-based pooled PCR tests:* Recommending the use of saliva-based PCR tests, particularly for their acceptability in school and kindergarten settings, should be considered. This method has shown promise in our study for increasing testing compliance, especially among vulnerable groups. However, decision-making on whether tests should be mandatory must be context-specific, considering pandemic dynamics.

*Other considerations:* Besides acceptance, sensitivity/specificity, testing time, invasiveness, and cost-effectiveness are crucial factors that should guide the selection of the appropriate test method.

1. Specific assistance for vulnerable groups

*Age-specific support:* For younger children, such as those in primary school, integrating external educational professionals to assist school staff can alleviate their workload. These professionals can explain tests in child-friendly terms and administer them efficiently to minimize discomfort.

*Support for children with mental health issues:* Tailored preparation and support are essential for children with mental health challenges, ensuring they feel comfortable and reassured during testing procedures.

1. Additional education and motivation

*Targeted educational campaigns:* Regions with higher proportions of parents with lower educational levels would benefit from enhanced educational campaigns about testing benefits and procedures.

*Testing ambassadors:* Introducing “testing ambassadors” within communities could effectively raise awareness and promote testing. These ambassadors, trained individuals from local communities, can advocate for testing benefits and provide guidance tailored to community needs (84).

These propositions aim to leverage our study findings to optimize testing strategies, fostering broader acceptance and effectiveness of public health measures by providing a clear path forward based on empirical findings, ensuring relevance and applicability in real-world public health settings. Implementing these recommendations could contribute to mitigating the impact of infectious diseases and enhancing overall community health resilience.

## Data availability statement

The datasets presented in this article are not readily available because the ethics committee did not grant permission to share study data with third parties or to upload data in anonymized form. Requests to access the datasets should be directed to stephan.bender@uk-koeln.de.

## Ethics statement

The studies involving humans were approved by Ethik-Kommission der Albert-Ludwigs-Universität Freiburg; Ethikkommission der Medizinischen Fakultät der Universität zu Köln. The studies were conducted in accordance with the local legislation and institutional requirements. Written informed consent for participation in this study was provided by the participants’ legal guardians/next of kin.

## Author contributions

JL: Methodology, Formal analysis, Writing – review & editing. CK: Writing – review & editing, Writing – original draft. SK: Writing – review & editing, Validation, Data curation. HW: Writing – review & editing. TL: Writing – review & editing, Supervision. EB: Writing – original draft, Writing – review & editing. SB: Writing – review & editing, Writing – original draft, Supervision, Resources, Methodology, Formal analysis, Data curation, Conceptualization.
