# Supplementary material for: Influence parental- and child-related factors on the acceptance of SARS-CoV-2 test methods in schools and daycare facilities
Source: Front Public Health. 2024 Jul 18;12:1264019. doi: 10.3389/fpubh.2024.1264019 (PMC11291219; doi:10.3389/fpubh.2024.1264019)
Supplement: Supplementary file 1 [file Table_1.docx]

*Supplementary Table S1. Descriptive comparison of the parent ratings (school grades) for the different test methods*

| Method | Mean | Standard error | CI | n |
| --- | --- | --- | --- | --- |
| Lolli PCR | 1.828 | 0.034 | 1.761; 1.895 | 1414 |
| Nasal swab antigen | 3.050 | 0.057 | 2.938; 3.162 | 501 |
| Saliva-antigen | 3.302 | 0.195 | 2.920; 3.685 | 43 |

| *Supplementary Table S2.*  *Model without* *Age* | |  |  | |  |
| --- | --- | --- | --- | --- | --- |
| effect | SQ | FG | MQ | F | *p* |
| constant | 540.581 | 1 | 540.5810 | 415.1977 | <0.001 |
| SDQ total | 50.530 | 1 | 50.5300 | 38.8100 | <0.001 |
| education | 7.790 | 1 | 7.7900 | 5.9831 | 0.015 |
| Covid test method | 244.822 | 1 | 244.8220 | 188.0376 | <0.001 |
| sex | 0.047 | 1 | 0.0470 | 0.0361 | 0.849 |
| vaccination status | 368.196 | 2 | 184.0980 | 141.3980 | <0.001 |
| error | 1702.996 | 1308 | 1.3020 |  |  |
| R² | 0.342 | | | | |
| adjusted R² | 0.339 | | | | |

| *Supplementary Table S3.*  *Model without* *SDQ* | |  |  | |  |
| --- | --- | --- | --- | --- | --- |
| effect | SQ | FG | MQ | F | *p* |
| constant | 587.546 | 1 | 587.5464 | 438.2671 | <0.001 |
| age | 11.887 | 1 | 11.8869 | 8.8668 | 0.003 |
| education | 0.005 | 1 | 0.0047 | 0.0035 | 0.953 |
| Covid test method | 249.407 | 1 | 249.4074 | 186.0398 | <0.001 |
| vaccination status | 429.216 | 2 | 214.6082 | 160.0822 | <0.001 |
| sex | 0.836 | 1 | 0.8358 | 0.6234 | 0.430 |
| error | 1753.521 | 1308 | 1.3406 |  |  |
| R² | 0.323 | | | | |
| adjusted R² | 0.319 | | | | |
|  | | | | | |

| *Supplementary Table S4.*  *Model without parental education* | |  |  | |  |
| --- | --- | --- | --- | --- | --- |
| effect | SQ | FG | MQ | F | *p* |
| constant | 693.706 | 1 | 693.7062 | 530.6891 | <0.001 |
| age | 0.001 | 1 | 0.0009 | 0.0007 | 0.979 |
| SDQ total | 55.153 | 1 | 55.1526 | 42.1920 | <0.001 |
| Covid test method | 242.140 | 1 | 242.1398 | 185.2383 | <0.001 |
| vaccination status | 377.283 | 2 | 188.6413 | 144.3116 | <0.001 |
| sex | 0.045 | 1 | 0.0449 | 0.0344 | 0.853 |
| error | 1712.406 | 1308 | 1.3072 |  |  |
| R² | 0.339 | | | | |
| adjusted R² | 0.336 | | | | |
|  | | | | | |

| *Supplementary Table S5.*  *Model without* *sex* | |  |  | |  |
| --- | --- | --- | --- | --- | --- |
| effect | SQ | FG | MQ | F | *p* |
| constant | 379.895 | 1 | 379.8948 | 291.7764 | <0.001 |
| age | 0.018 | 1 | 0.0183 | 0.0141 | 0.906 |
| SDQ total | 51.332 | 1 | 51.3324 | 39.4256 | <0.001 |
| education | 7.832 | 1 | 7.8318 | 6.0152 | 0.0143 |
| Covid test method | 240.692 | 1 | 240.6915 | 184.8620 | <0.001 |
| vaccination status | 353.334 | 2 | 176.6672 | 135.6884 | <0.001 |
| error | 1703.025 | 1308 | 1.3020 |  |  |
| R² | 0.342 | | | | |
| adjusted R² | 0.339 | | | | |
| \| *Supplementary Table S6.*  *Model without* *vaccination status* \| \|  \|  \| \|  \| \| --- \| --- \| --- \| --- \| --- \| --- \| \| effect \| SQ \| FG \| MQ \| F \| *p* \| \| constant \| 435.459 \| 1 \| 435.4592 \| 277.1982 \| <0.001 \| \| age \| 14.844 \| 1 \| 14.8435 \| 9.4489 \| 0.002 \| \| SDQ total \| 126.389 \| 1 \| 126.3894 \| 80.4551 \| <0.001 \| \| education \| 29.585 \| 1 \| 29.5849 \| 18.8327 \| <0.001 \| \| Covid test method \| 317.011 \| 1 \| 317.0111 \| 201.7983 \| <0.001 \| \| sex \| 0.011 \| 1 \| 0.0108 \| 0.0069 \| 0.934 \| \| error \| 2056.348 \| 1308 \| 1.5709 \|  \|  \| \| R² \| 0.206 \| \| \| \| \| \| adjusted R² \| 0.202 \| \| \| \| \| \|  \| \| \| \| \| \| | | | | | |

| *Supplementary Table S7.*  *Model without* *test method* | |  |  | |  |
| --- | --- | --- | --- | --- | --- |
| Effect | SQ | FG | MQ | F | *p* |
| constant | 299.514 | 1 | 299.5141 | 201.5566 | <0.001 |
| age | 4.123 | 1 | 4.1235 | 2.7749 | 0.096 |
| SDQ total | 59.234 | 1 | 59.2341 | 39.8613 | <0.001 |
| education | 8.982 | 1 | 8.9822 | 6.0445 | 0.014 |
| sex | 0.022 | 1 | 0.0216 | 0.0145 | 0.904 |
| vaccination status | 429.665 | 2 | 214.8325 |  |  |
| error | 1943.694 | 1308 | 1.4860 |  |  |
| R² | 0.249 | | | | |
| adjusted R² | 0.246 | | | | |
